# Expression of a Subset of Heat Stress Induced Genes of *Mycobacterium tuberculosis* Is Regulated by 3',5'-Cyclic AMP

**DOI:** 10.1371/journal.pone.0089759

**Published:** 2014-02-28

**Authors:** Eira Choudhary, William Bishai, Nisheeth Agarwal

**Affiliations:** 1 Vaccine and Infectious Disease Research Center, Translational Health Science and Technology Institute, Gurgaon, Haryana, India; 2 Center for Tuberculosis Research, Johns Hopkins University School of Medicine, Baltimore, Maryland, United States of America; Tulane University, United States of America

## Abstract

*Mycobacterium tuberculosis* (Mtb) secretes excess of a second messenger molecule, 3',5'-cyclic AMP (cAMP), which plays a critical role in the survival of Mtb in host macrophages. Although Mtb produces cAMP in abundance, its exact role in the physiology of mycobacteria is elusive. In this study we have analyzed the expression of 16 adenylate cyclases (ACs) and kinetics of intracellular cAMP levels in Mtb during *in vitro* growth under the regular culture conditions, and after exposure to different stress agents. We observed a distinct expression pattern of these ACs which is correlated with intracellular cAMP levels. Interestingly cAMP levels are significantly elevated in Mtb following heat stress, whereas other stress conditions such as oxidative, nitrosative or low pH do not affect intracellular cAMP pool *in vitro*. A significant increase in expression by >2-fold of five ACs namely Rv1647, Rv2212, Rv1625c, Rv2488c and Rv0386 after heat stress further suggested that cAMP plays an important role in controlling Mtb response to heat stress. In the light of these observations, effect of exogenous cAMP on global gene expression profile was examined by using microarrays. The microarray gene expression analysis demonstrated that cAMP regulates expression of a subset of heat stress-induced genes comprising of *dnaK*, *grpE*, *dnaJ*, and *Rv2025c*. Further we performed electrophoretic mobility shift assay by using cAMP-receptor protein of Mtb (CRP^M^), which demonstrated that CRP^M^ specifically recognizes a sequence _−301_ AGCGACCGTCAGCACG_−286_ in 5'-untranslated region of *dnaK*.

## Introduction

*Mycobacterium tuberculosis* (Mtb), the causative agent of disease tuberculosis (TB) has evolved a clever strategy of intoxicating host macrophages by secreting a signaling molecule, 3',5'-cyclic adenosine monophosphate (cAMP) [1], [2]. Cyclic AMP is continuously produced by Mtb during *in vitro* growth [3], probably due to presence of multiple adenylate cyclases (ACs). Genome sequence of Mtb reveals the presence of 16 ACs in Mtb H_37_Rv strain, 10 of which have been biochemically characterized *in vitro* [4]–[7]. Fusion of class III adenylyl cyclase catalytic region with different domains adds versatility to these multiple ACs. The first AC of Mtb which was characterized by *in vitro* biochemical assays is Cya which is encoded by a gene *Rv1625c* [8], [9]. Structurally each monomeric subunit of Cya homodimer contains six transmembrane regions and a catalytic domain, which corresponds to one half of the mammalian adenylyl cyclases [10]. Other functionally characterized ACs of Mtb are: pH-sensing Rv1264 which contains an autoinhibitory N-terminal domain [11]–[12]; Rv1318c, Rv1319c, Rv1320c and Rv3645 containing membrane anchored HAMP (present in Histidine kinases, Adenylate cyclases, Methyl accepting proteins and Phosphatases) region [13]; Rv0386 whose adenylyl cyclase catalytic domain is fused to an AAA-ATPase and a helix-turn-helix DNA-binding domain [14]; Rv1647 and Rv2212 which attain complete activity only in the presence of detergent and unsaturated fatty acids, respectively [15], [16]; and Rv1900c which forms asymmetric homodimers [17]. Cyclic AMP is also secreted into host cells during infection which perturbs signaling pathways and affects bacterial persistence and killing by host macrophages [1], [2].

In prokaryotes cAMP activates the function of a transcription factor known as cAMP-receptor protein or CRP which recognizes a specific sequence in the 5'-untranslated region (5'-UTR) and subsequently regulates the mRNA synthesis of candidate genes [4]. *In silico* analysis predicts 10 putative cNMP-binding proteins in Mtb [4]; two of these proteins encoded by *Rv3676* (known as cAMP-receptor protein of *M. tuberculosis*, CRP^M^) and *Rv1675c* (annotated as Cmr, for cAMP and macrophage regulator) function as cAMP-responsive transcription factors that regulate expression of multiple genes by direct binding to their promoter regions [18]–[24]. In addition to regulating mycobacterial pathogenesis other important biological processes are also regulated by this signaling molecule. Exogenous cAMP stimulates the expression of galactokinase in the presence of glutamate and galactose in *Mycobacterium smegmatis* (Msm), which is otherwise not induced by galactose alone [25]. In *M. bovis* BCG cAMP regulates the expression of five proteins namely Rv1265, Rv2971, GroEL2, PE_PGRS6a, and malate dehydrogenase [26]. Very recently it is shown that cAMP plays a role in acetylation of stress proteins and acetyl-CoA synthetase [27]–[29], which suggests that cAMP is critical in functioning of central metabolic pathways of Mtb.

Though Mtb produces significant concentration of cAMP which is also secreted into extracellular environment [30], [31], direct role of cAMP in the physiology of Mtb is lacking. By using a systematic approach in this study we measured the intracellular cAMP levels and expression of ACs in Mtb during its *in vitro* growth in regular culture medium as well as under different stress conditions. By performing a whole genome microarray analysis, we studied the effect of cAMP on global gene expression profile of Mtb. Further, direct effect of cAMP on the expression of candidate genes was validated by performing electrophoretic mobility shift assay (EMSA). Our results demonstrate that in Mtb cAMP levels are significantly elevated after heat stress which in-turn regulates the expression of a subset of heat stress-induced genes encoding chaperones DnaK, GrpE and DnaJ respectively, by facilitating the direct binding of CRP^M^ to the promoter region of *dnaK* operon.

## Results

### Analysis of intracellular cAMP levels in Mtb during *in vitro* growth

**Figure 1:**
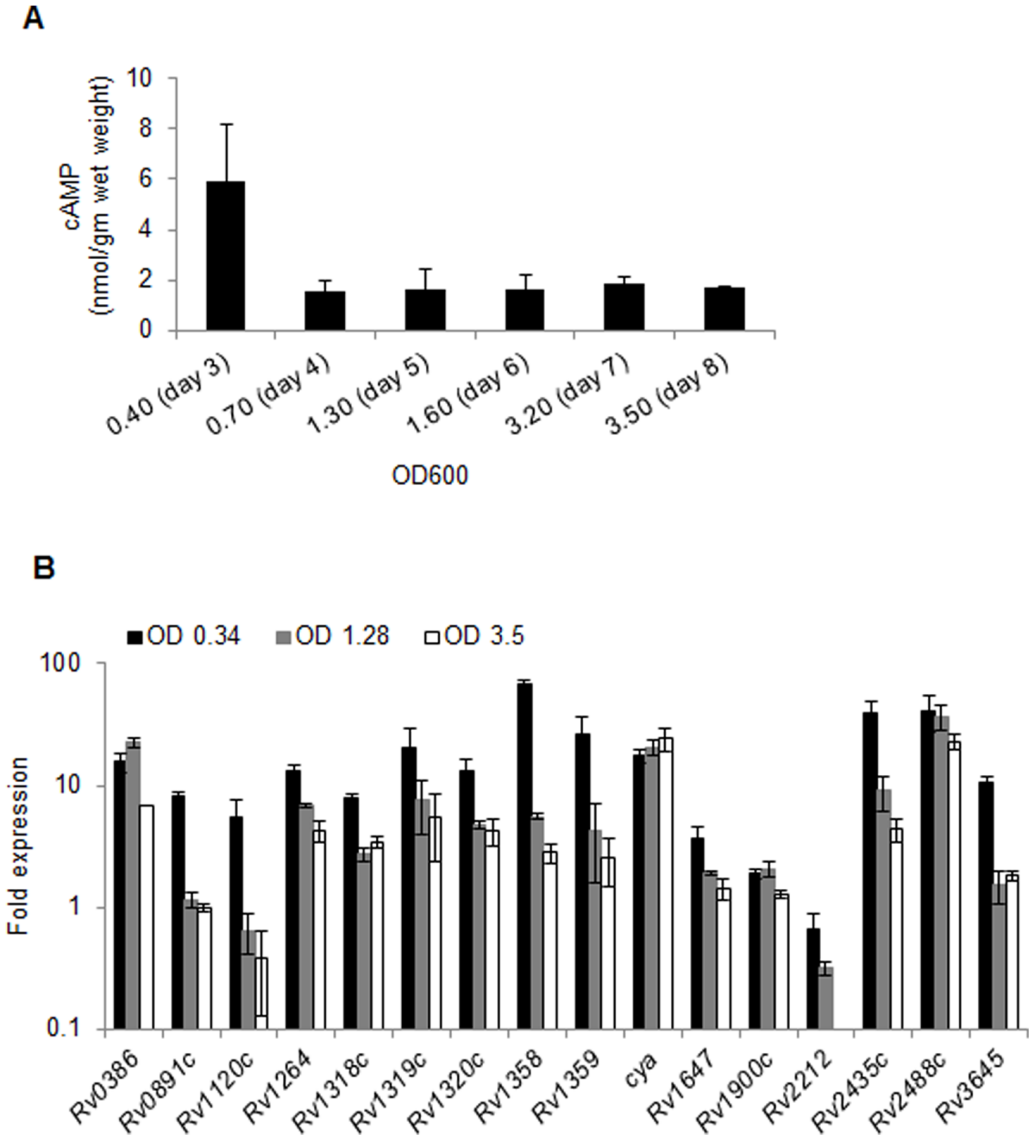
Kinetics of intracellular cAMP levels and expression of ACs in Mtb during *in vitro* growth. A) Estimation of intracellular cAMP levels in Mtb. Wild-type Mtb CDC1551 was cultured in Middlebrook 7H9 broth supplemented with OADC, glycerol, and tween-80 at 37°C with shaking and growth was monitored at regular intervals by measuring optical density of the cultures at 600 nm (OD_600_). An aliquot of culture suspension was used at each time point up to 8 days for cAMP measurement, as described in materials and methods, and final cAMP concentration was calculated as nmol/gm wet weight. Data are the averages of two independent experiments and the mean values ± standard deviations are shown. B) Analysis of the expression of AC-encoding genes at different time points during *in vitro* growth of Mtb. Total RNA was isolated from Mtb CDC1551 cultures at regular intervals as shown in the graph. Quantitative RT-PCR (qRT-PCR) was performed, and the relative expression of each gene was calculated using values of *sigA* transcript for normalizing the RNA amounts and day-1 expression value as a control. The graph represents an average of two independent experiments and the mean values ± standard deviations are shown.

Cyclic AMP is known to exert an array of regulatory functions which advocates that the cellular concentration of cAMP must itself be subject to control by culture conditions. Here we estimated intracellular cAMP levels in pathogenic Mtb grown in 7H9 culture medium supplemented with 1x OADC (oleic acid, albumin, dextrose and catalase), 0.5% glycerol and 0.02% tween-80, at different growth stages. Lysates were prepared by boiling the bacterial pellets in 0.1 M HCl to avoid degradation of cyclic nucleotides during extraction by phosphodiesterase (PDE) of Mtb, and cAMP was measured by ELISA as described in materials and methods. Our results demonstrated that Mtb exhibited maximum intracellular cAMP at day3 post-inoculation when the optical density at 600 nm (OD_600_) of bacterial culture was 0.4. Subsequent growth on day 4 (OD_600_ of 0.70) resulted in sharp decline in the intracellular cAMP pool by ∼3.5 folds and this level remained constant for next four days of growth when cultures reached to stationary phase (Fig. 1A). By ELISA, it was estimated that cAMP concentrations were 5.9 nmol/gm wet weight on day 3, and 1.6–1.8 nmol/gm wet weight on days 4–8 respectively (Fig. 1A).

### Expression analysis of Mtb ACs by real-time quantitative reverse-transcription PCR (qRT-PCR)

Cellular concentration of cAMP can be regulated at the level of expression and/or activity of AC and the PDE, or by a change in the rate of cAMP export [6], [32]–[34]. Although intracellular cAMP levels are significantly altered in Mtb, we observed that the extracellular cAMP pool remains constant over eight days of *in vitro* growth (data not shown). Since it is challenging to determine intracellular enzymatic activities of multiple ACs or PDEs, we focused on studying the expression profiles of mycobacterial ACs by qRT-PCR at various OD_600_ (Fig. 1B). Transcript levels of each of the 16 AC-encoding genes at designated OD_600_ were compared with their respective expression levels at day 1 post-inoculation in wild-type Mtb when the OD_600_ of culture was 0.1. Figure 1B shows that majority of AC-encoding genes except *Rv1647*, *Rv1900c* and *Rv2212* were overexpressed by ≥5-fold when OD_600_ of culture reached to 0.34 on day 3. In contrast, transcripts of *Rv1647* and *Rv1900c* were upregulated by 3.6 and 1.9-fold, respectively, whereas *Rv2212* exhibited moderate reduction at OD_600_ of 0.34. Interestingly, at day 5 when OD_600_ of Mtb culture reached to 1.28, expression of most of the genes except *Rv0386*, *cya*, *Rv1900c* and *Rv2488c* was reduced by 2–12 folds compared to their respective expression levels at OD_600_ of 0.34 on day 3. Contrary to these, *Rv0386*, *cya*, *Rv1900c* and *Rv2488c* either maintained or displayed moderate increase in expression at OD_600_ of 1.28. Further growth of mycobacteria to OD_600_ of 3.5 at day 8 post-inoculation resulted in moderate decrease in expression of few genes that included *Rv0386*, *Rv1358* and *Rv2435c* by 3.2-, 1.97- and 2.07-folds, respectively, while others were expressing at levels similar to OD_600_ 1.28 (Fig. 1B). In contrast, *Rv2212* transcript was not detected at OD_600_ of 3.5. These results are in line with the profile of cellular cAMP concentrations in Mtb which suggests that intracellular cAMP levels vary in proportion to the expression of ACs in Mtb (Fig. 1).

### Effect of various stresses on intracellular cAMP levels and expression of Mtb ACs

Upon infection, virulent mycobacteria encounter stringent antimicrobial response within the host organism. However, tubercle bacilli are resistant to killing by host and persist for decades in the host tissues [35]. For their survival, mycobacterial pathogens sense and respond to exogenous stress conditions by modulating the expression of key genes. It is known that cAMP-associated transcription factors CRP^M^ and Cmr regulate the expression of several genes in Mtb which play important roles in Mtb–host interactions [18]–[24]. Interestingly, intracellular cAMP levels in mycobacteria increase dramatically after infection to macrophages [33]. These observations led us to hypothesize that Mtb must be equipped to modulate its cAMP pool under different stress conditions imposed by the host. Therefore we monitored the intracellular cAMP levels in Mtb after its exposure to different stress conditions that bacteria encounter during host infection.

**Figure 2:**
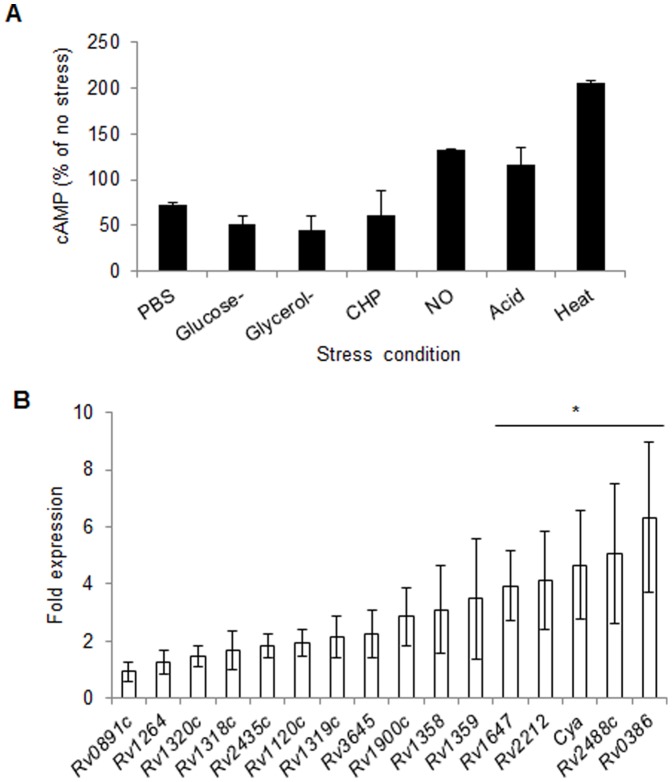
Effect of various stresses on intracellular cAMP levels and expression of ACs in Mtb. A) Estimation of cAMP in Mtb during *in vitro* growth under different stresses. Wild-type culture suspension in Middlebrook 7H9 broth at OD_600_ of 0.4 was exposed to different stresses as described earlier [36] and intracellular cAMP levels were determined by ELISA. Bar graph represents cAMP level in each of the stressed samples as percentage of un-treated control. Data are the averages of three independent experiments and the mean values ± standard deviations are shown. B) Expression analysis of the AC-encoding genes following thermal stress in Mtb. The fold-expression, as measured by qRT-PCR, indicates the ratio of *sigA*-normalized gene expression levels in Mtb exposed to 42 °C relative to those in the Mtb grown at 37°C. The graph represents an average of three experiments. Error bars indicate standard deviations and asterisk denotes *p* ≤0.05.

Wild-type Mtb cells were exposed to nutrient starvation (PBS), glucose deficiency, glycerol deficiency, oxidative stress (cumene hydroperoxide, CHP), nitosative stress (DETA/NO), acid stress (pH 4.5) and heat stress (42°C), as described earlier [36]. Subsequently, cells were lysed and cAMP was measured in the lysates by ELISA. As shown in figure 2A, a moderate decrease in intracellular cAMP levels was observed after nutrient starvation and oxidative stress, whereas deficiency of any of the two carbon sources resulted in ∼2-fold reduction in the intracellular cAMP concentration (Fig. 2A). In contrast, a mild increase in intracellular cAMP level was seen in the cells after nitrosative and acid stresses. Interestingly, of these stresses, maximum effect was observed after heat-stress, which caused >2-fold increase in intracellular cAMP level under these culture conditions.

In order to assess whether increase in cAMP level after the heat-stress is related to the expression of ACs, we performed the qRT-PCR to measure mRNA levels of AC-encoding genes in Mtb following the heat-shock treatment. Expression level of individual gene was obtained after normalization with the level of a housekeeping gene *sigA*, which remained unaltered under these conditions. Figure 2B depicts that the mRNA levels of 5 out of 16 AC-encoding genes (*Rv1647*, *Rv2212*, *cya*, *Rv2488c* and *Rv0386*) are increased by >2-fold after heat-stress (*p* ≤0.05). Taken together, these observations indicate that Mtb responds to heat-shock exposure by inducing the expression of certain ACs which results in elevated cAMP-levels in the cell.

### Identification of cAMP-regulon by microarray

**Figure 3:**
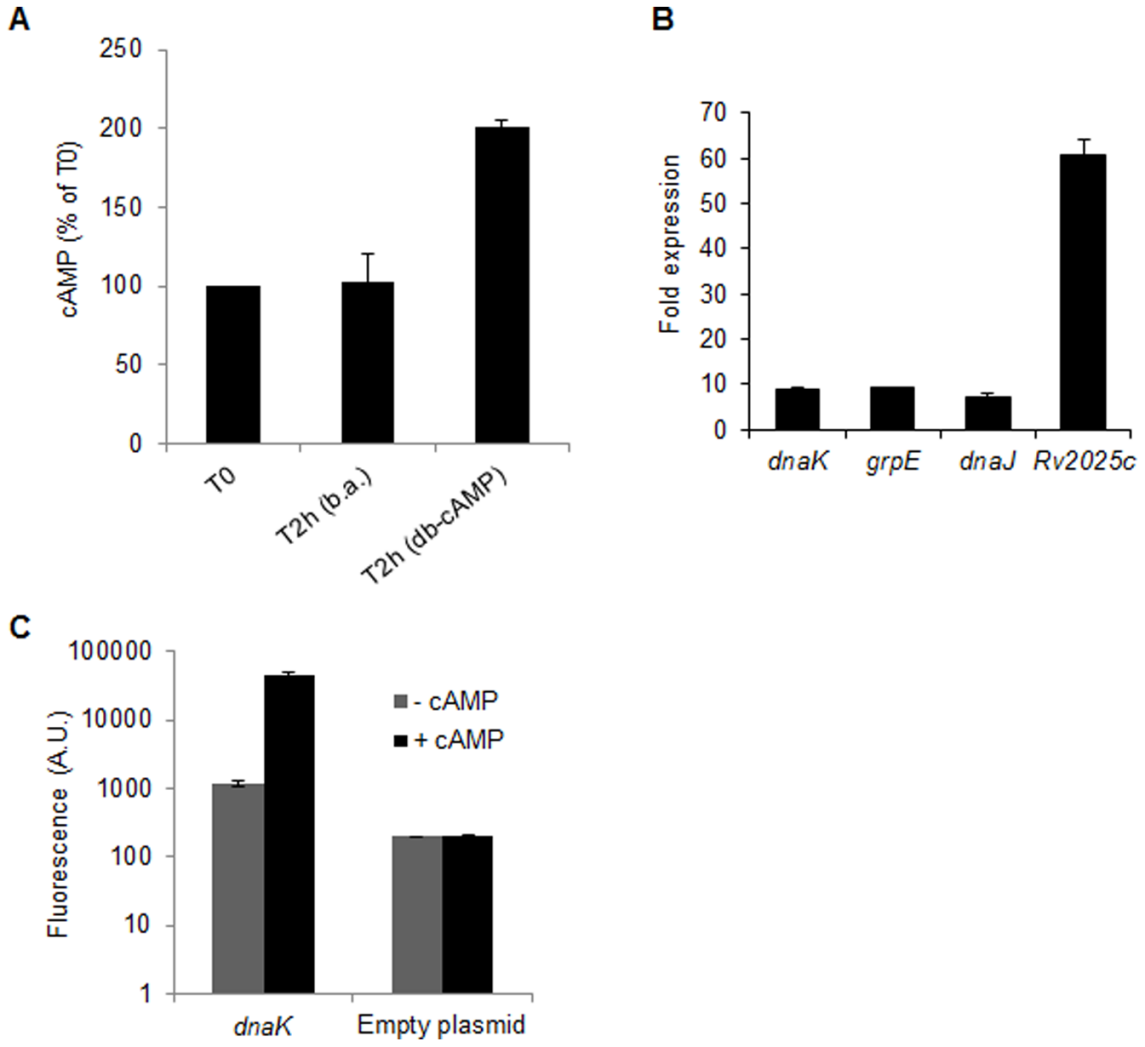
Effect of cAMP on global gene expression profile of Mtb. A) Effect of db-cAMP treatment on intracellular cAMP levels in Mtb. *In vitro* grown cultures of Mtb were treated with 20 mM butyric acid (b.a.) or db-cAMP for 2 hrs, followed by intracellular cAMP estimation by ELISA. Shown are the intracellular cAMP levels after 2 hrs of treatment (T2h) compared to that in untreated cells at zero hour time point (T0). B) Validation of differential expression of genes in microarray analysis by qRT-PCR. Genes upregulated in the db-cAMP-treated Mtb relative to the butyric acid-treated sample by microarray analysis were evaluated by qRT-PCR. The fold-expression indicates the ratio of *sigA*-normalized gene expression levels in the db-cAMP-treated strain relative to those in the butyric acid-treated Mtb. C) Effect of db-cAMP on *in vivo* activity of *dnaK* promoter. *In vivo* activity of *dnaK* promoter was assessed by measuring β-gal expression in Msm harboring pSD5B-*dnaK* (*dnaK*) using β-gal fluorescent substrate (C2FDG). Promoter activity was determined in mycobacterial cultures treated with 10 mM db-cAMP (+cAMP) or butyric acid (-cAMP) for 2 hrs. The empty plasmid pSD5B was used as a control. Data represent an average of three independent experiments and error bars indicate standard deviations.

To identify cAMP-regulated genes of Mtb, wild-type Mtb CDC1551cultures were treated with di-butyryl cAMP (db-cAMP) and the expression of genes was compared with butyric acid-treated control samples by whole genome microarrays as described in materials and methods. Before proceeding to microarray experiments, intracellular cAMP levels were measured in both the samples by ELISA, which indicated a 2-fold increase in cAMP levels after 2 hrs of incubation with 20 mM db-cAMP (Fig. 3A). In contrast, there was no change in intracellular cAMP levels after incubation with 20 mM butyric acid for 2 hrs, which suggests that db-cAMP specifically elevates intracellular cAMP pool by 2-fold (Fig. 3A). A total of three hybridization experiments were performed with three independent RNA preparations, and the results were analyzed statistically. Expression level of individual gene was measured after normalization with the level of *sigA*, which was not changed under these conditions. Genes that were differentially expressed by >2.0-fold (*p*≤ 0.05) in db-cAMP-treated samples are shown in table 1. The microarray results demonstrated that a total of 7 genes were upregulated and 5 genes were downregulated by ≥2-fold after treatment with db-cAMP, in comparison to butyric acid-treated samples (Table 1). The most abundant transcripts were those of a subset of heat stress-induced genes [37] comprising of *Rv2025c, dnaK, grpE and dnaJ*. Further, differences in expression of these genes between db-cAMP-treated and butyric acid-treated samples by microarray were 9.8±4.4, 3.7±0.6, 2.9±0.9, and 3.2±0.7 respectively (Table 1). These differences were more prominent in qRT-PCR which exhibited 60.9±3.2, 8.9±0.4, 9.1±0.1, and 7.3±0.6-fold induction of *Rv2025c, dnaK, grpE and dnaJ* respectively after db-cAMP treatment (Fig. 3B). Moreover, expression levels of these genes were significantly higher than of cAMP-regulated *whiB1* [20], which exhibited 1.4±0.18-fold induction under these conditions. Other genes that exhibited notable induction in expression levels were *Rv0264c* (2.9±1.6-fold), *Rv1057* (2.3±0.2-fold), and *Rv1330c* (*pncB1*, 2±1-fold). In contrast, five genes that include *Rv0146*, *mmpS5*, *fadD9*, *hupB*, and *Rv3830c* were significantly downregulated by 5.9, 2.1, 5.1, 2.0 and 8.5-folds respectively, in db-cAMP-treated Mtb samples (Table 1).

**Table 1:** Differentially expressed genes in Mtb treated with db-cAMP relative to butyric acid treated bacteria.

Gene product*^b^*	Locus	Change in expression (fold)	SD (fold)	*P* value
CHP	*Rv0146*	–5.9	0.0	0.01
CHP	*Rv0264c*	2.9	1.6	0.02
DnaK	*Rv0350*	3.7	0.6	0.02
GrpE	*Rv0351*	2.9	0.9	0.05
DnaJ	*Rv0352*	3.2	0.7	0.03
MmpS5	*Rv0677c*	–2.1	0.2	0.00
CHP	*Rv1057*	2.3	0.2	0.04
CHP	*Rv1330c*	2.0	1.0	0.03
CMP	*Rv2025c*	9.8	4.4	0.02
FadD9	*Rv2590*	–5.1	0.1	0.04
HupB	*Rv2986c*	–2.0	0.0	0.02
TRP	*Rv3830c*	–8.5	0.0	0.03

Expression of 3924 genes of Mtb was compared between the cells treated with db-cAMP and butyric acid. An average of data from three independent experiments (*p*≤ 0.05) is included in the table. Genes that exhibit ≥2-fold difference in expression levels by whole-genome microarray are shown**.** SD: standard deviation. ***^b^***CHP, CMP and TRP denote conserved hypothetical protein, conserved membrane protein and transcription regulatory protein, respectively.

Next, we examined whether expression of these genes is altered in mycobacteria due to direct effect of cAMP on their promoter activity. A 450bp long DNA sequence corresponding to 5'-UTR of *dnaK* was cloned upstream to the *lacZ* in promoter probe plasmid pSD5B [20] and the recombinant plasmid pSD5B-*dnaK* was transformed into Msm. Expression of *lacZ* was subsequently monitored in the presence or absence of db-cAMP in Msm::pSD5B-*dnaK*. As shown in figure 3C, in the absence of db-cAMP (-cAMP), Msm::pSD5B-*dnaK* exhibited significant β-gal activity in comparison to Msm containing empty plasmid. Interestingly, addition of db-cAMP to bacterial cultures (+cAMP) resulted in ∼40-fold increase in activity of *dnaK* promoter, whereas there was no effect of db-cAMP on the basal expression levels of *lacZ* in empty plasmid containing control strain (Fig. 3C).

These results thus clearly demonstrate that cAMP acts as a regulator of the expression of multiple genes including a subset of heat stress-induced genes of Mtb.

### Identification of CRP^M^-recognition sequence in 5'-UTR of cAMP-regulon

The CRP of *E. coli* binds to a 16bp sequence TGTGA-N6-TCACA in the 5'-UTR of cAMP-regulon. Similar to *E. coli* the CRP of Mtb (CRP^M^) was also shown to regulate expression of key Mtb genes that bear the similar sequences in their 5'-UTR [18]–[20], [22], [23]. In order to understand whether cAMP-mediated expression of genes in our study is due to direct binding of CRP^M^ to their respective 5'-UTRs, we analyzed 350bp sequences upstream to the start codon of each of the 10 open reading frames (ORFs) by using the TubercuList database (http://genolist.pasteur.fr/TubercuList/) to identify the putative CRP^M^ consensus sequences bearing two mismatches in either of the left (TGTGA) or right (TCACA) arms. Since *dnaK* is transcribed in operon with *grpE* and *dnaJ* [37], we omitted the upstream sequences to *grpE* and *dnaJ* in our analysis. As shown in table 2 except *hupB*, all other ORFs exhibited putative CRP^M^-recognition sequences in their 5'-UTR. These observations suggest that cAMP may regulate the expression of these genes by facilitating the binding of cAMP-activated CRP^M^ to their promoter region.

**Table 2:** Analysis of putative CRP-recognition sequences in 5'-UTRs of cAMP-regulon of Mtb.

Locus (Rv/annotation)	Sequence*^a^*	Position from Start codon
*Rv0146*	tgtcgaggctttcacc	–325
*Rv0146*	tttcaccatgaacaca	–316
*Rv0146*	tgacaccggcatcacg	–45
*Rv0146*	agcgactcggtttaga	–266
*Rv0146*	tgtccaggcgttgacc	–244
*Rv0146*	cgagaccgtccgcacc	–103
*Rv0264c*	gctgatctggatgacc	–300
*Rv0350/dnaK*	agcgaccgtcagcacg	–301
*Rv0350/dnaK*	cgttagcatgctcagt	–135
*Rv0677c/mmpS5*	tttcactgtactctga	–81
*Rv0677c/mmpS5*	tgttcgacgaattcca	–149
*Rv0677c/mmpS5*	tctgaaatctgtgacg	–70
*Rv1057*	cgtgacctaggtaaca	–248
*Rv1057*	tcagaatttggtcgct	–208
*Rv1330c*	tgcggaccgcgtcgga	–21
*Rv1330c*	agtcacgtagctcatc	–233
*Rv1330c*	tggcatgcggctcgct	–276
*Rv2025c*	tctgagcaagctcagc	–246
*Rv2025c*	tgcgtatgaatgcaga	–82
*Rv2590/fadD9*	agtgaggggctggaca	–206
*Rv2590/fadD9*	cgtcatcattttgacc	–285
*Rv2590/fadD9*	cgtgccgcatctcaca	–133
*Rv3830c*	tgcgcgcagtctcgcc	–72
*Rv3830c*	tgtcaatgttgacaga	–42
*Rv3830c*	tttgtcaatgttgaca	–44

Three hundred fifty base pair long DNA sequences upstream to the respective ORFs were analyzed to identify CRP^M^-recognition sequence. ***^a^***The underlined sequences represent putative CRP^M^-recognition sequences (TGTGA-N6-TCACA, with two mismatches in any of the arms) in the 5'-UTR of the respective genes.

### EMSA to validate direct binding of CRP^M^- to 5'-UTR of *dnaK* operon

**Figure 4:**
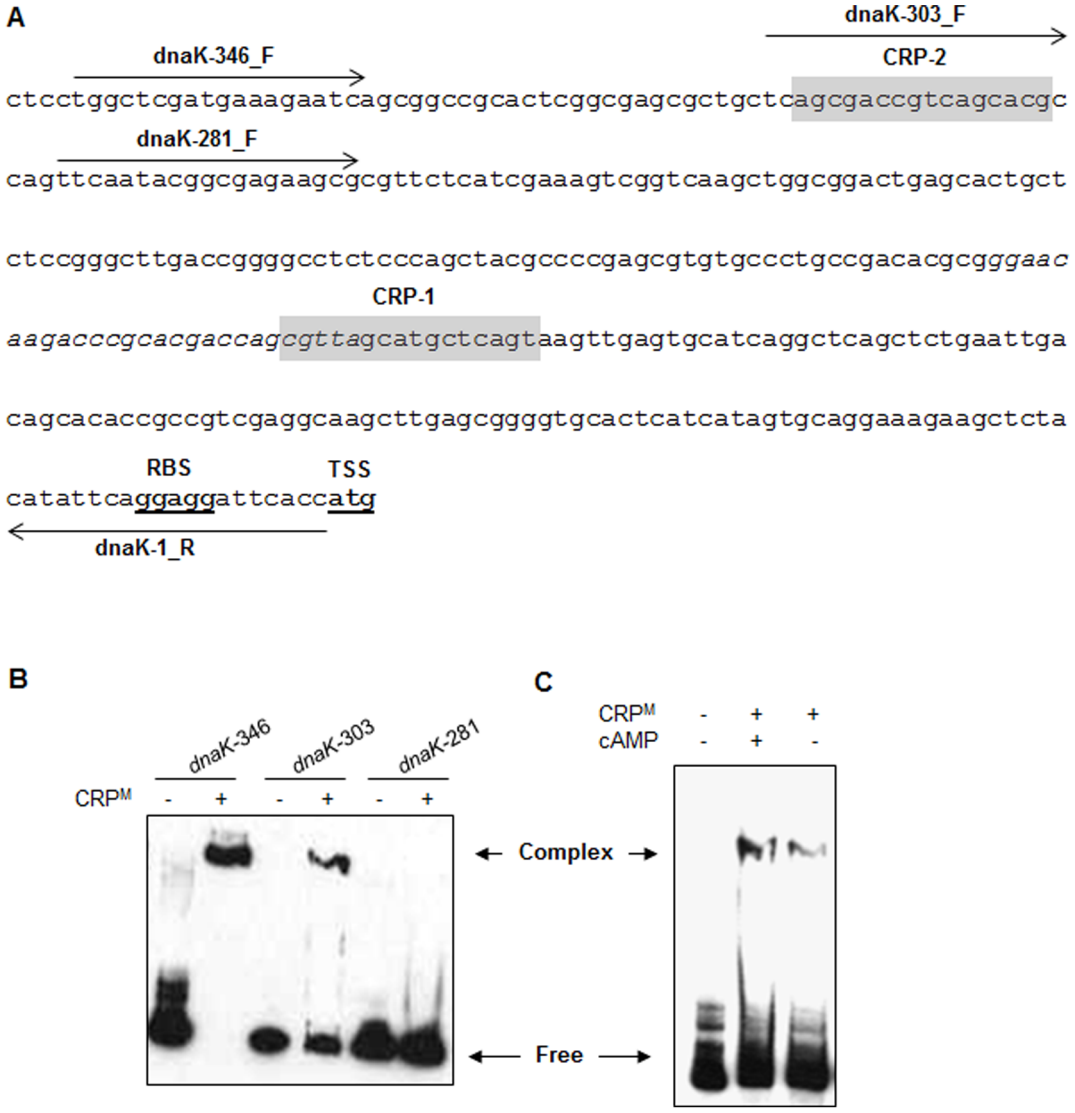
Identification of CRP-binding motif in promoter region of *dnaK* operon by EMSA. A) Sequence of the 5'-UTR of *dnaK* operon. The putative ribosome-binding site (RBS), and the translation start site (TSS) are boldfaced and underlined. The putative CRP-binding sites are shaded, whereas the SigH-recognition sequence is shown in italics. Positions and directions of the primers that were used for PCR amplification of DNA fragments shown in (B) are marked by horizontal arrows. B) Interaction of CRP^M^ with the 5'-UTR of *dnaK* operon. Biotin-labeled *dnaK* promoter fragments of various lengths, as shown, were incubated with CRP^M^ in the presence of 1 mM cAMP before separation of DNA-protein complexes by gel electrophoresis. Reaction mixtures containing promoter fragments but lacking the CRP^M^ were resolved in adjacent lanes as controls. C) Effect of cAMP on complex formation between CRP^M^ and *dnaK* promoter. Purified CRP^M^ pre-incubated with or without 1 mM cAMP for 30 min on ice, was subject to binding with full length *dnaK* promoter fragment (*dnaK*-346) for 15 min at 37°C before separation of CRP^M^–*dnaK* complexes by gel electrophoresis. Complex, CRP^M^–*dnaK* complex; Free, unbound DNA fragment.

Analysis of 5'-UTR of *dnaK* operon indicates the presence of two putative CRP^M^-consensus sequences. The first sequence CRP-1 (5'- CGTTAGCATGCTCAGT-3') is located between positions -135 and -120 from translation start codon, whereas the second sequence CRP-2 (5'- AGCGACCGTCAGCACG-3') is situated further upstream between positions –301 and –286 from translation start codon (Fig. 4A). A careful observation demonstrates that CRP-2 is located 127bp upstream to SigH-binding site which is positioned between –158 to –130 from translation stop codon (5'- GGGAACAAGACCCGCACGACCAGCGTTA-3'), whereas CRP-1 overlaps with SigH-recognition sequence in 5'-UTR of *dnaK* (Fig. 4A). Since *dnaK, grpE and dnaJ* were amongst the most abundant transcripts in db-cAMP-treated Mtb, we sought to determine if their expression is controlled by direct binding of CRP^M^ to the promoter sequence in the 5'-UTR. CRP^M^ was purified as earlier [20] and subjected to binding with DNA fragment comprising of sequence between –1 to –346 from translation start codon of *dnaK* (*dnaK*-346). As shown in figure 4B, CRP^M^ makes a specific complex with this sequence. Further deletion of 43bp from 5' end (*dnaK*-303) indicated that CRP^M^ continues to bind this sequence, albeit with ∼2-fold reduced affinity as assessed by the intensity of CRP^M^-DNA complex. On the other hand deletion of additional 22bp (*dnaK*-281) which resulted in complete loss of CRP-2 site located between –301 and –286, completely abolished the complex formation (Fig. 4B). These results thus establish that: i) regulation of *dnaK* expression is governed by direct binding of CRP^M^ to its 5'-UTR, ii) CRP^M^ binds at CRP-2 site and not at CRP-1 site in the *dnaK* promoter, and iii) binding of CRP^M^ to 5'-UTR of *dnaK* and its subsequent effect on mRNA expression is independent of SigH binding.

Presence of cAMP enhances binding of CRP^M^ to corresponding promoter DNA sequences [20], [23]. Hence, we analyzed if association of CRP^M^ to *dnaK* promoter is also influenced by cAMP. Binding reactions were carried out in the absence (apo-CRP^M^) or the presence of 1 mM cAMP, and the CRP^M^-DNA complexes were resolved on polyacrylamide gel as described in materials and methods. Figure 4C shows that presence of cAMP enhances the complex formation between CRP^M^ and *dnaK*-346 promoter fragment by 3-fold compared to that with apo-CRP^M^. These results thus clearly indicate that cAMP regulates the expression of *dnaK* operon by facilitating the binding of CRP^M^ to its promoter.

## Discussion

This study was designed to estimate the intracellular cAMP levels over a period of *in vitro* growth and its role in gene expression of Mtb. Cyclic AMP in bacteria was first reported half a century ago when the cyclic nucleotide was observed in the culture filtrate of *Bravibacterium liquefaciens* [38] and later in *E. coli* [39]. Subsequently occurrence of cAMP was reported in other bacteria including mycobacteria [3]. Later after discovery of cAMP in bacteria, it was studied that cAMP plays an important role in assimilation of sugar molecules by regulating a process called catabolite repression [40], [41]. Levels of cAMP keep changing as a function of glucose level in the cell [41]. Although in mycobacteria cAMP is not involved in carbon metabolism, variation in intracellular cAMP levels over a period of *in vitro* growth (Fig. 1A) indicates that cAMP levels are dynamic. A constant extracellular cAMP concentration rules out the prospect of differential export of cAMP to outside culture medium. Although effects of differential activities of ACs, PDEs and other associated factors on intracellular cAMP concentrations is not ruled out, a similar pattern of expression of 16 ACs and intracellular cAMP levels during *in vitro* growth of Mtb suggests that the relative abundance of ACs could be an important factor governing the intracellular cAMP levels in Mtb (Fig. 1A and B). While, the trigger(s) of sudden changes in expression of ACs and subsequent cellular cAMP levels at late growth stages of Mtb is yet to identify, the intracellular polyphosphates may be an important determinant of cellular cAMP levels [42]. Polyphosphates, generated by the activity of an enzyme known as polyphosphate kinase, is highly accumulated at the stationary phase as well as under different stress conditions in Mtb [43], and are known to inhibit the activity of ACs [42]. These information warrant further studies to analyze the effect of polyphosphates on intracellular cAMP levels.

Microbial pathogens including Mtb are adapted to survive under diverse stress conditions. Effects of cAMP on bacterial responses to cold-shock [44], expression of stress proteins [45] and RpoS, a late stationary sigma factor which also regulates oxidative stress response [46], [47] indicate that cAMP is an important determinant of the bacterial response to variety of stresses other than carbon metabolism in many pathogenic bacteria. Recently it was shown that cAMP regulates the response of uropathogenic *E. coli* to nitrosative, oxidative and acid stresses, and affects its virulence [48]. In contrast to these organisms, intracellular cAMP in Mtb was significantly stimulated after the heat stress whereas other stresses such as oxidative, nitrosative or acid exhibited a milder effect (Fig. 2A). Response to heat stress is an adaptive response to a sudden increase in ambient temperature, which involves a group of proteins such as chaperons, proteases, and regulatory factors commonly known as heat shock proteins [37]. Transcriptionally heat shock proteins are expressed in Mtb by involving an extracellular function sigma protein, SigH [49]. Upregulated expression of 5 ACs after the heat stress and presence of SigH-consensus sequences in the upstream promoter region of their respective ORFs (Fig. 2B and Table 3) together suggest that apparent induction of cAMP pool under these conditions may be due to differential transcription of mycobacterial AC-encoding genes by SigH. Parallel to these observations, induction of genes encoding primary heat shock proteins DnaK, GrpE, DnaJ1 and Rv2025c by exogenous cAMP indicates that a subset of heat shock proteins are expressed in Mtb under the effect of cAMP (Table 1 and Fig. 3B-C). Absence of CRP/Cmr-recognition sequences in *sigH* promoter and lack of its induction by cAMP together suggest that cAMP-driven expression of heat shock genes may not be directly controlled by SigH. Alternatively our results propose that cAMP regulates the expression of these genes by facilitating the binding of CRP^M^ to their respective promoter sequences. This was further confirmed by the presence of CRP-consensus sequence in the upstream promoter regions of 9 out of 10 ORFs that were differentially expressed in Mtb after cAMP-treatment (Table 2), and by EMSA studies with CRP^M^ which specifically recognized a 16-bp sequence _-301_ AGCGACCGTCAGCACG_−286_ in the 5'-UTR of *dnaK* operon, in a cAMP-dependent manner (Fig. 4).

**Table 3:** Identification of putative SigH-recognition sequences in the 5'-UTRs of AC-encoding genes of Mtb.

Locus (Rv/annotation)	Sequence*^a^*	Position from Start codon
*Rv0386*	cggaaatccaccgtccggtggcgtcgcttc	–98
*Rv1625c/cya*	cgcaacatctcggccaggtccatgcggatg	–335
*Rv1647*	ggcaacgcggtgaccggcttcctgtttg	–311
*Rv2212*	cgaaactcgcacccagctcgcgatggcggtc	–114
*Rv2488c*	cggcaccgcccacgatgcggtcgtgtcggttc	–152

Three hundred fifty base pair long DNA sequences upstream to the AC-encoding genes were analyzed to identify SigH-recognition sequences. ***^a^***The underlined sequences represent putative SigH-recognition sequences (c/gGGAAc-N_17–21_-c/gGTTc/g) in the 5'-UTR of the respective genes.

DnaK is a 70 kDa chaperone which is conserved in almost all living organisms. In Mtb DnaK is one of the most abundant proteins, which regulates Mtb virulence [50]. DnaK plays a crucial role in protecting bacteria from thermal or oxidative stress that cause partial unfolding and possible aggregation of cellular proteins. It has ability to bind the hydrophobic residues of partially unfolded proteins, exposed by stress which subsequently prevents them from aggregating, and allows them to refold. Cyclic AMP is universally present in mycobacteria and its levels are further elevated under stress conditions. Regulation of DnaK expression by cAMP indicates that this secondary messenger molecule is critical in regulating mycobacterial response to stress.

## Materials and Methods

### Bacterial strains, culture conditions and plasmid

For culturing of Mtb CDC1551, Middlebrook 7H9 broth supplemented with 1x OADC, 0.02% tween-80 and 0.5% glycerol, and Middlebrook 7H10 agar supplemented with 1x OADC and 0.5% glycerol were used. Msm mc^2^155 was cultured in 7H9 broth containing 0.02% tween-80 and 0.5% glycerol. Bacteria were grown at 37°C with (in 7H9 broth) or without (on 7H10-agar) shaking at 200 rotations per minute. For culturing in glucose-free medium, synthetic supplement containing oleic acid, albumin and saline equivalent to 1x concentration were added to 7H9 medium containing 0.02% tween-80 and 0.5% glycerol. Bacteria were treated with different stress agents as described earlier [36]. The promoter probe vector pSD5B [20] was kindly provided by Dr. Anil Tyagi, University of Delhi South Campus, New Delhi, India. To determine the *in vivo* activity of *dnaK* promoter in mycobacteria, the 450bp region upstream to translational start site of *dnaK* ORF was amplified using primer pairs dnak-450F (5'-GGGTCTAGAGCACCGTTGGCCCGTTCGATG-3') and dnak-1R (5'-CCCTCTAGAGGTGAATCCTCCTGAATATGTAG-3'), and cloned at *Xba* I site in pSD5B plasmid upstream to the *lacZ* resulting in pSD5B-*dnaK*. The pSD5B-*dnaK* plasmid harboring *dnaK* promoter in sense orientation was subsequently electroporated in Msm and the recombinant Msm::pSD5B-*dnaK* strains were selected on Middlebrook 7H10 agar containing 0.5% glycerol and 25 mg/L kanamycin.

### Estimation of cAMP by ELISA

Intracellular cAMP determination in Mtb was performed with clarified cell lysate after heat lysis of cell pellets in 0.1 M HCl. Cyclic AMP was estimated by ELISAs using Enzyme Immunoassay Kits (Assay Designs Inc., Ann Arbor, MI) according to the manufacturer’s instructions. The intracellular cAMP levels in Mtb were estimated as nmol/gm wet weight.

### RNA extraction

Total RNA was isolated from Mtb by using the TRIzol suspension according to manufacturer’s instructions (Invitrogen Corporation, Carlsbad, CA).

### Real-time quantitative reverse transcription PCR

RNA isolated from the bacterial cultures was subjected to treatment with RNase-free DNase I (Ambion) to remove traces of contaminating DNA. Subsequently, absence of DNA in the RNA preparations was verified by 30 cycles of PCR followed by ethidium-bromide-stained agarose gel analysis before proceeding with reverse transcription of the RNAs. Complementary DNA (cDNA) synthesized from total RNA was subjected to real-time quantitative reverse transcription PCR typically as described earlier [36].

### Microarray analysis

To analyze the effect of cAMP on the expression of mycobacterial genes, Mtb was cultured to OD_600_ of 0.6 and pelleted down by centrifugation at 6000 x *g* for 10min at 4°C. Culture pellets were washed twice with 1 x phosphate buffered saline, pH 7.4 (PBS) and suspended in 1/10^th^ volume of 7H9 medium containing either of the 20 mM butyric acid (control) or di-butyryl cAMP (db-cAMP, test). After 2 hrs of incubation at 37°C in roller bottles, cells were pelleted down, washed 3 x with PBS and RNA was isolated as described above. For probe preparation, cDNA was synthesized from 5 µg of total RNA from the test and control strains of Mtb and labeled with Cy3 or Cy5 (GE Healthcare). Microarray slides were prepared by using 70-mer oligos encompassing the entire genome of Mtb (GEO accession number GSE54289) and hybridization of labeled cDNAs was performed typically as described earlier [36]. RNA samples were prepared from 3 biological replicates. Slide scanning and data analysis was performed as previously described [36].

### β-Galatosidase assay

Promoter activity of *dnaK* was measured in Msm::pSD5B-*dnaK* by fluorescent based detection of *lacZ* expression, as described earlier [51]. Briefly, Msm::pSD5B-*dnaK* cultures were grown in 7H9 broth medium containing kanamycin (25 mg/L) to an OD_600_ of 1.0, washed twice with 7H9 medium and resuspended in 7H9 medium. The OD_600_ of culture suspension was adjusted to 1.0, and the cultures were incubated with either 10 mM db-cAMP or 10 mM butyric acid for 2 hrs. Hundred microliter of culture suspensions were taken into 3 separate wells of a 96-well black fluoroplate (Greiner Bio-One). Fluorescent β-gal substrate, 5-acetylamino-FDG (C2FDG) (Life Technologies) was subsequently added to a final concentration of 33 µM into each well and β-gal activity was estimated by measuring the fluorescence after 1 hr of incubation using a spectrofluorometer (Biotek) with an excitation of 485±20 nm and emission of 528±20 nm. A similar experiment was performed with Msm containing empty plasmid, pSD5B as control.

### EMSA

DNA probes for EMSA analysis were synthesized by PCR amplification of the desired regions of the *dnaK* promoter, using biotin-labeled oligonucleotides as the primers (Fig. 4A). The amplicons were purified from agarose gels and used for gel-shift experiments. The EMSA experiments were performed typically as described earlier [20].

## References

[1] Agarwal N, Lamichhane G, Gupta R, Nolan S, Bishai WR (2009) Cyclic AMP intoxication of macrophages by a Mycobacterium tuberculosis adenylate cyclase. Nature 460: 98–102. 19516256 10.1038/nature08123

[2] Agarwal N, Bishai WR (2009) cAMP signaling in Mycobacterium tuberculosis. Indian J Exp Biol 47: 393–400. 19634702

[3] Padh H, Venkitasubramanian TA (1977) Adenosine 3', 5'-monophosphate in mycobacteria. Life Sci 20: 1273–1280. 191715 10.1016/0024-3205(77)90502-1

[4] McCue LA, McDonough KA, Lawrence CE (2000) Functional classification of cNMP-binding proteins and nucleotide cyclases with implications for novel regulatory pathways in Mycobacterium tuberculosis. Genome Res 10: 204–219. 10673278 10.1101/gr.10.2.204

[5] Shenoy AR, Sivakumar K, Krupa A, Srinivasan N, Visweswariah SS (2004) A survey of nucleotide cyclases in actinobacteria: unique domain organization and expansion of the class III cyclase family in Mycobacterium tuberculosis. Comp Funct Genomics 5: 17–38. 18629044 10.1002/cfg.349 PMC2447327

[6] Shenoy AR, Visweswariah SS (2006) New messages from old messengers: cAMP and mycobacteria. Trends Microbiol 14: 543–550. 17055275 10.1016/j.tim.2006.10.005

[7] Shenoy AR, Visweswariah SS (2006) Mycobacterial adenylyl cyclases: biochemical diversity and structural plasticity. FEBS Lett 580: 3344–3352. 16730005 10.1016/j.febslet.2006.05.034

[8] Guo YL, Seebacher T, Kurz U, Linder JU, Schultz JE (2001) Adenylyl cyclase Rv1625c of Mycobacterium tuberculosis: a progenitor of mammalian adenylyl cyclases. EMBO J 20: 3667–3675. 11447108 10.1093/emboj/20.14.3667 PMC125536

[9] Reddy SK, Kamireddi M, Dhanireddy K, Young L, Davis A, et al (2001) Eukaryotic-like adenylyl cyclases in Mycobacterium tuberculosis H37Rv: cloning and characterization. J Biol Chem 276: 35141–35149. 11431477 10.1074/jbc.M104108200

[10] Ketkar AD, Shenoy AR, Ramagopal UA, Visweswariah SS, Suguna K (2006) A structural basis for the role of nucleotide specifying residues in regulating the oligomerization of the Rv1625c adenylyl cyclase from M. tuberculosis. J Mol Biol 356: 904–916. 16403515 10.1016/j.jmb.2005.12.017

[11] Linder JU, Schultz A, Schultz JE (2002) Adenylyl cyclase Rv1264 from Mycobacterium tuberculosis has an autoinhibitory N-terminal domain. J Biol Chem 277: 15271–15276. 11839758 10.1074/jbc.M200235200

[12] Tews I, Findeisen F, Sinning I, Schultz A, Schultz JE, et al (2005) The structure of a pH-sensing mycobacterial adenylyl cyclase holoenzyme. Science 308: 1020–1023. 15890882 10.1126/science.1107642

[13] Linder JU, Hammer A, Schultz JE (2004) The effect of HAMP domains on class IIIb adenylyl cyclases from Mycobacterium tuberculosis. Eur J Biochem 271: 2446–2451. 15182360 10.1111/j.1432-1033.2004.04172.x

[14] Castro LI, Hermsen C, Schultz JE, Linder JU (2005) Adenylyl cyclase Rv0386 from Mycobacterium tuberculosis H37Rv uses a novel mode for substrate selection. Febs J 272: 3085–3092. 15955067 10.1111/j.1742-4658.2005.04722.x

[15] Shenoy AR, Sreenath NP, Mahalingam M, Visweswariah SS (2005) Characterization of phylogenetically distant members of the adenylate cyclase family from mycobacteria: Rv1647 from Mycobacterium tuberculosis and its orthologue ML1399 from M. leprae. Biochem J 387: 541–551. 15500449 10.1042/BJ20041040 PMC1134983

[16] Abdel Motaal A, Tews I, Schultz JE, Linder JU (2006) Fatty acid regulation of adenylyl cyclase Rv2212 from Mycobacterium tuberculosis H37Rv. FEBS J 273: 4219–4228. 16925585 10.1111/j.1742-4658.2006.05420.x

[17] Sinha SC, Wetterer M, Sprang SR, Schultz JE, Linder JU (2005) Origin of asymmetry in adenylyl cyclases: structures of Mycobacterium tuberculosis Rv1900c. EMBO J 24: 663–673. 15678099 10.1038/sj.emboj.7600573 PMC549627

[18] Bai G, McCue LA, McDonough KA (2005) Characterization of Mycobacterium tuberculosis Rv3676 (CRPMt), a cyclic AMP receptor protein-like DNA binding protein. J Bacteriol 187: 7795–7804. 16267303 10.1128/JB.187.22.7795-7804.2005 PMC1280308

[19] Rickman L, Scott C, Hunt DM, Hutchinson T, Menendez MC, et al (2005) A member of the cAMP receptor protein family of transcription regulators in Mycobacterium tuberculosis is required for virulence in mice and controls transcription of the rpfA gene coding for a resuscitation promoting factor. Mol Microbiol 56: 1274–1286. 15882420 10.1111/j.1365-2958.2005.04609.x PMC2964915

[20] Agarwal N, Raghunand TR, Bishai WR (2006) Regulation of the expression of whiB1 in Mycobacterium tuberculosis: role of cAMP receptor protein. Microbiology 152: 2749–2756. 16946269 10.1099/mic.0.28924-0

[21] Akhter Y, Tundup S, Hasnain SE (2007) Novel biochemical properties of a CRP/FNR family transcription factor from Mycobacterium tuberculosis. Int J Med Microbiol 297: 451–457. 17702648 10.1016/j.ijmm.2007.04.009

[22] Gazdik MA, Bai G, Wu Y, McDonough KA (2009) Rv1675c (cmr) regulates intramacrophage and cyclic AMP-induced gene expression in Mycobacterium tuberculosis-complex mycobacteria. Mol Microbiol 71: 434–448. 19040643 10.1111/j.1365-2958.2008.06541.x PMC2845544

[23] Stapleton M, Haq I, Hunt DM, Arnvig KB, Artymiuk PJ, et al (2010) Mycobacterium tuberculosis cAMP receptor protein (Rv3676) differs from the Escherichia coli paradigm in its cAMP binding and DNA binding properties and transcription activation properties. J Biol Chem 285: 7016–7027. 20028978 10.1074/jbc.M109.047720 PMC2844151

[24] Bai G, Schaak DD, Smith EA, McDonough KA (2011) Dysregulation of serine biosynthesis contributes to the growth defect of a Mycobacterium tuberculosis crp mutant. Mol Microbiol 82: 180–198. 21902733 10.1111/j.1365-2958.2011.07806.x PMC3785234

[25] Raychaudhuri S, Basu M, Mandal NC (1998) Glutamate and cyclic AMP regulate the expression of galactokinase in Mycobacterium smegmatis. Microbiology 144 ( Pt 8): 2131–2140. 10.1099/00221287-144-8-2131 9720034

[26] Gazdik MA, McDonough KA (2005) Identification of cyclic AMP-regulated genes in Mycobacterium tuberculosis complex bacteria under low-oxygen conditions. J Bacteriol 187: 2681–2692. 15805514 10.1128/JB.187.8.2681-2692.2005 PMC1070381

[27] Nambi S, Basu N, Visweswariah SS (2010) cAMP-regulated protein lysine acetylases in mycobacteria. J Biol Chem 285: 24313–24323. 20507997 10.1074/jbc.M110.118398 PMC2915667

[28] Xu H, Hegde SS, Blanchard JS (2011) Reversible acetylation and inactivation of Mycobacterium tuberculosis acetyl-CoA synthetase is dependent on cAMP. Biochemistry 50: 5883–5892. 21627103 10.1021/bi200156t PMC3125470

[29] Nambi S, Gupta K, Bhattacharyya M, Ramakrishnan P, Ravikumar V, et al (2013) Cyclic AMP-dependent Protein Lysine Acylation in Mycobacteria Regulates Fatty Acid and Propionate Metabolism. J Biol Chem 288: 14114–14124. 23553634 10.1074/jbc.M113.463992 PMC3656268

[30] Lee CH (1977) Identification of adenosine 3',5'-monophosphate in Mycobacterium smegmatis. J Bacteriol 132: 1031–1033. 200600 10.1128/jb.132.3.1031-1033.1977 PMC235607

[31] Lee CH (1979) Metabolism of cyclic AMP in non-pathogenic Mycobacterium smegmatis. Arch Microbiol 120: 35–37. 218515 10.1007/BF00413269

[32] Shenoy AR, Sreenath N, Podobnik M, Kovacevic M, Visweswariah SS (2005) The Rv0805 gene from Mycobacterium tuberculosis encodes a 3',5'-cyclic nucleotide phosphodiesterase: biochemical and mutational analysis. Biochemistry 44: 15695–15704. 16313172 10.1021/bi0512391

[33] Bai G, Schaak DD, McDonough KA (2009) cAMP levels within Mycobacterium tuberculosis and Mycobacterium bovis BCG increase upon infection of macrophages. FEMS Immunol Med Microbiol 55: 68–73. 19076221 10.1111/j.1574-695X.2008.00500.x PMC3222459

[34] Bai G, Knapp GS, McDonough KA (2011) Cyclic AMP signalling in mycobacteria: redirecting the conversation with a common currency. Cell Microbiol 13: 349–358. 21199259 10.1111/j.1462-5822.2010.01562.x PMC3785248

[35] Forrellad MA, Klepp LI, Gioffre A, Sabio y Garcia J, Morbidoni HR, et al (2013) Virulence factors of the Mycobacterium tuberculosis complex. Virulence 4: 3–66. 23076359 10.4161/viru.22329 PMC3544749

[36] Agarwal N, Woolwine SC, Tyagi S, Bishai WR (2007) Characterization of the Mycobacterium tuberculosis sigma factor SigM by assessment of virulence and identification of SigM-dependent genes. Infect Immun 75: 452–461. 17088352 10.1128/IAI.01395-06 PMC1828396

[37] Stewart GR, Wernisch L, Stabler R, Mangan JA, Hinds J, et al (2002) Dissection of the heat-shock response in Mycobacterium tuberculosis using mutants and microarrays. Microbiology 148: 3129–3138. 12368446 10.1099/00221287-148-10-3129

[38] Okabayashi T, Ide M, Yoshimoto A (1963) Excretion of adenosine-3',5'-phosphate in the culture broth of Brevibacterium liquefaciens. Arch Biochem Biophys 100: 158–159. 13939741 10.1016/0003-9861(63)90046-8

[39] Makman RS, Sutherland EW (1965) Adenosine 3',5'-Phosphate in Escherichia Coli. J Biol Chem 240: 1309–1314. 14284741

[40] Perlman R, Pastan I (1968) Cyclic 3'5-AMP: stimulation of beta-galactosidase and tryptophanase induction in E. coli. Biochem Biophys Res Commun 30: 656–664. 4966929 10.1016/0006-291x(68)90563-9

[41] Ullmann A, Monod J (1968) Cyclic AMP as an antagonist of catabolite repression in Escherichia coli. FEBS Lett 2: 57–60. 11946268 10.1016/0014-5793(68)80100-0

[42] Guo YL, Mayer H, Vollmer W, Dittrich D, Sander P, et al (2009) Polyphosphates from Mycobacterium bovis--potent inhibitors of class III adenylate cyclases. FEBS J 276: 1094–1103. 19154349 10.1111/j.1742-4658.2008.06852.x

[43] Singh R, Singh M, Arora G, Kumar S, Tiwari P, et al (2013) Polyphosphate Deficiency in Mycobacterium tuberculosis Is Associated with Enhanced Drug Susceptibility and Impaired Growth in Guinea Pigs. J Bacteriol 195: 2839–2851. 23585537 10.1128/JB.00038-13 PMC3697247

[44] Ohsaka Y, Ohgiya S, Hoshino T, Ishizaki K (2001) Cold-stimulated increase in a regulatory subunit of cAMP-dependent protein kinase in human hepatoblastoma cells. DNA Cell Biol 20: 667–673. 11749725 10.1089/104454901753340659

[45] Jeong KC, Baumler DJ, Kaspar CW (2006) dps expression in Escherichia coli O157:H7 requires an extended -10 region and is affected by the cAMP receptor protein. Biochim Biophys Acta 1759: 51–59. 16574257 10.1016/j.bbaexp.2006.02.001

[46] Barth E, Gora KV, Gebendorfer KM, Settele F, Jakob U, et al (2009) Interplay of cellular cAMP levels, {sigma}S activity and oxidative stress resistance in Escherichia coli. Microbiology 155: 1680–1689. 19372151 10.1099/mic.0.026021-0 PMC2848814

[47] Cheng Y, Sun B (2009) Polyphosphate kinase affects oxidative stress response by modulating cAMP receptor protein and rpoS expression in Salmonella typhimurium. J Microbiol Biotechnol 19: 1527–1535. 20075614

[48] Donovan GT, Norton JP, Bower JM, Mulvey MA (2013) Adenylate cyclase and the cyclic AMP receptor protein modulate stress resistance and virulence capacity of uropathogenic Escherichia coli. Infect Immun 81: 249–258. 23115037 10.1128/IAI.00796-12 PMC3536135

[49] Raman S, Song T, Puyang X, Bardarov S, Jacobs WR Jr, et al (2001) The alternative sigma factor SigH regulates major components of oxidative and heat stress responses in Mycobacterium tuberculosis. J Bacteriol 183: 6119–6125. 11567012 10.1128/JB.183.20.6119-6125.2001 PMC99691

[50] Stewart GR, Snewin VA, Walzl G, Hussell T, Tormay P, et al (2001) Overexpression of heat-shock proteins reduces survival of Mycobacterium tuberculosis in the chronic phase of infection. Nat Med 7: 732–737. 11385512 10.1038/89113

[51] Rowland B, Purkayastha A, Monserrat C, Casart Y, Takiff H, et al (1999) Fluorescence-based detection of lacZ reporter gene expression in intact and viable bacteria including Mycobacterium species. FEMS Microbiol Lett 179: 317–325. 10518732 10.1111/j.1574-6968.1999.tb08744.x

